# Association between plasma proBDNF levels and cognitive impairment in patients with alcohol dependence: a case–control and longitudinal study

**DOI:** 10.3389/fpsyt.2026.1835592

**Published:** 2026-06-10

**Authors:** Hongying Pan, Le Zhang, Yuhang Liang, Wei Wang, Chen Yu, Yunyun Li, Li Wu, Hongyan Liu, Li Zhou

**Affiliations:** The Affiliated Mental Health Center of Kunming Medical University, Yunnan Kunming, China

**Keywords:** alcohol dependence, biomarker, cognitive impairment, executive function, ProBDNF

## Abstract

**Background:**

Alcohol dependence is frequently accompanied by cognitive impairment. Brain-derived neurotrophic factor (BDNF) signaling plays a critical role in synaptic plasticity, while the precursor form, proBDNF, has been increasingly implicated in neurodegenerative and psychiatric disorders. However, the association between plasma proBDNF levels and cognitive impairment in alcohol dependence remains unclear.

**Methods:**

Eighty male patients with alcohol dependence and forty-two matched healthy controls were enrolled. Plasma proBDNF levels were measured via enzyme-linked immunosorbent assay (ELISA). Cognitive function was assessed using the Mini-Mental State Examination (MMSE), the Modified Wisconsin Card Sorting Test (M-WCST), and the Verbal Fluency Test (VFT). Forty-one patients were reassessed after four weeks of abstinence. Group comparisons and correlation analyses were performed.

**Results:**

Patients with alcohol dependence exhibited significantly elevated plasma proBDNF levels and impaired cognitive performance compared with controls. Plasma proBDNF levels were positively correlated with alcohol consumption severity, and linked to global cognitive deficits alongside nuanced executive performance variations. After four weeks of abstinence, plasma proBDNF levels decreased and cognitive performance improved; however, changes in proBDNF were weakly associated with cognitive recovery.

**Conclusions:**

Elevated plasma proBDNF levels are associated with alcohol dependence severity and cognitive impairment, suggesting that proBDNF may serve as a peripheral biomarker reflecting the dynamic neurocognitive status in alcohol dependence.

## 1 Introduction

Alcohol dependence is a chronic relapsing disorder that imposes a substantial burden on global public health and is frequently accompanied by persistent cognitive impairment. Chronic alcohol exposure has been shown to induce neuronal apoptosis, mitochondrial dysfunction, and oxidative stress, ultimately disrupting synaptic integrity and neuroplasticity (1, 2). Clinically, individuals with alcohol dependence often exhibit deficits across multiple cognitive domains, particularly in executive function, attention, and memory, significantly impairing daily functioning and treatment outcomes (3, 4). Accumulating evidence indicates that alcohol-related cognitive deficits may persist even after prolonged abstinence. Meta-analyses and longitudinal studies have demonstrated widespread and enduring impairments in executive control, information processing speed, and working memory among individuals with alcohol dependence, although partial recovery may occur with sustained abstinence (4–6). The degree of cognitive impairment is influenced by drinking patterns, cumulative alcohol exposure, and age, suggesting that neurobiological alterations induced by alcohol are complex and heterogeneous (7–9). Despite increasing recognition of alcohol-induced cognitive impairment, the underlying molecular mechanisms remain incompletely understood.

Brain-derived neurotrophic factor (BDNF) is a key regulator of neuronal survival, synaptic plasticity, and cognitive function. Since its initial purification from mammalian brain tissue (10), BDNF has been extensively studied for its role in learning, memory, and adaptive neuroplasticity. At the synaptic level, BDNF signaling is essential for long-term potentiation (LTP), dendritic spine maintenance, and cortical network stability (11–13). More recent evidence has further highlighted that BDNF expression, secretion, and activity-dependent release are tightly regulated and critically linked to synaptic plasticity and memory-related processes (14). Experimental studies have demonstrated that ethanol exposure alters BDNF expression and disrupts BDNF-mediated neuroprotective effects, thereby exacerbating neuronal vulnerability and cognitive decline (1, 15, 16). Importantly, BDNF is synthesized as a precursor protein (proBDNF) that can be proteolytically cleaved into mature BDNF (mBDNF), and these two isoforms exert distinct and often opposing biological effects. According to the yin–yang model of neurotrophin signaling, mBDNF preferentially binds to the TrkB receptor to promote neuronal survival, synaptic strengthening, and LTP, whereas proBDNF activates the p75 neurotrophin receptor (p75 NTR) to induce synaptic weakening, long-term depression (LTD), and neuronal apoptosis (13, 17, 18). The balance between proBDNF and mBDNF is therefore critical for maintaining synaptic homeostasis and cognitive integrity (11, 19).

Emerging evidence suggests that dysregulation of the proBDNF/mBDNF system may play a crucial role in neuropsychiatric and neurodegenerative disorders. Elevated proBDNF levels have been reported in Alzheimer’s disease and other conditions characterized by synaptic loss and cognitive decline (19, 20). Experimental studies further indicate that proBDNF suppresses hippocampal neurogenesis, inhibits neural stem cell proliferation, and impairs learning and memory through activation of p75 NTR signaling pathways (18, 20, 21). Conversely, neutralization of proBDNF or inhibition of p75 NTR signaling has been shown to ameliorate cognitive deficits in various disease models (21). In the context of alcohol dependence, increasing attention has been directed toward the role of BDNF signaling. Both animal and human studies have reported alterations in circulating and central BDNF levels associated with alcohol consumption, withdrawal, and relapse vulnerability (8, 15, 22, 23). However, findings regarding total BDNF levels in alcohol dependence have been inconsistent, with reports of increased, decreased, or unchanged concentrations, possibly due to methodological differences and the lack of differentiation between proBDNF and mBDNF (24–27).

Despite these advances, clinical evidence linking plasma proBDNF levels to cognitive impairment in alcohol dependence remains limited. Most existing studies have focused on total BDNF without assessing its precursor form, and few have systematically examined the relationship among proBDNF, alcohol-related clinical variables, and domain-specific cognitive function. Therefore, the present study aimed to investigate plasma proBDNF levels in patients with alcohol dependence and matched healthy controls, and to examine the associations among proBDNF, alcohol consumption severity, and cognitive function, providing an empirical basis for understanding the link between plasma proBDNF levels and cognitive impairment in alcohol-dependent patients.

## 2 Methods

### 2.1 Participants

This study recruited patients with alcohol dependence who were consecutively admitted to the Department of Alcohol and Drug Dependence Treatment (now renamed the Department of Addiction Medicine), the Affiliated Mental Health Center of Kunming Medical University between January 2022 and December 2022. Alcohol dependence was diagnosed according to the International Classification of Diseases, 10th Revision (ICD-10), and diagnoses were independently confirmed by at least two attending psychiatrists or senior clinicians. During the study period, a total of 409 male patients aged 18–55 years with alcohol dependence were admitted to the department. Following rigorous screening against the inclusion and exclusion criteria, 80 patients were enrolled in the baseline group, representing an enrollment rate of 19.6%. A group of 42 healthy male volunteers matched for age, sex, and educational level were recruited from routine physical examinations at the same institution as the healthy control group. Healthy controls had no history of alcohol abuse or dependence and reported no alcohol consumption during the three months preceding enrollment. Self-reported drinking histories and clinical interviews were supplemented by routine physical examination data; gamma-glutamyl transferase (GGT) and mean corpuscular volume (MCV) values were within normal reference ranges for all included controls. Among the patients with alcohol dependence, 41 individuals completed a four-week inpatient abstinence treatment and were reassessed at follow-up. These participants constituted the longitudinal treatment group. The four-week period was selected to reflect early abstinence during inpatient treatment. All participants were of Han ethnicity and aged between 18 and 55 years.

Inclusion and exclusion criteria as follow:

Inclusion criteria for patients with alcohol dependence were (1): meeting ICD-10 diagnostic criteria for alcohol dependence (2); Alcohol Use Disorders Identification Test (AUDIT) score ≥ 8 (3); no prior systematic treatment for the current episode of alcohol dependence; and (4) provision of written informed consent by the participant or legal guardian.

Exclusion criteria included (1): diagnosis of Wernicke encephalopathy or Korsakoff syndrome (2); comorbid psychiatric disorders such as schizophrenia, bipolar disorder, or major depressive disorder (3); comorbid dependence on substances other than nicotine (4); use of sedative or hypnotic medications within one month prior to enrollment (5); history of major neurological, cardiovascular, or systemic diseases; and (6) severe visual impairment or cognitive impairment that prevented completion of neuropsychological testing.

Because the longitudinal follow-up was conducted in an inpatient rehabilitation setting, no parallel continued-drinking comparison group was available.

The study protocol was approved by the Ethics Committee of the Affiliated Mental Health Center of Kunming Medical University (Approval No. YNJS-20211110-001), and all procedures were conducted in accordance with the Declaration of Helsinki. Notably, all participants were voluntarily admitted and provided independent written informed consent; no involuntarily admitted patients were included in the present study.

### 2.2 Treatment protocol

Patients underwent a standardized four-week inpatient detoxification program. Withdrawal management followed a symptom-triggered approach based on CIWA-Ar scores; benzodiazepines (diazepam 15–40 mg/d, oxazepam 45–90 mg/d, or lorazepam 2–6 mg/d) were clinically titrated and typically tapered and discontinued within the initial 7–10 days. All participants received oral vitamin B complex upon admission. During the first 5–7 days, intramuscular thiamine (200 mg/d) was administered, followed by oral vitamin B1 (10 mg, tid) for the duration of the study. Low-dose second-generation antipsychotics (e.g., quetiapine 25–50 mg/d or olanzapine 2.5–5 mg/d) were occasionally used as adjunctive therapy for agitation or insomnia. Notably, no anti-craving medications (e.g., naltrexone) were prescribed. Additionally, all patients participated in weekly group psychotherapy sessions incorporating elements of cognitive-behavioral therapy and motivational interviewing.

### 2.3 Clinical assessments

Demographic and clinical information, including age, educational level, duration of alcohol use, and average daily alcohol intake during the previous month, was collected using standardized questionnaires and medical records. Alcohol use severity was assessed using the Alcohol Use Disorders Identification Test (AUDIT), a 10-item screening tool evaluating alcohol consumption, drinking behaviors, and alcohol-related problems. The Severity of Alcohol Dependence Questionnaire (SADQ) was administered to evaluate the degree of alcohol dependence. Withdrawal symptom severity was evaluated using the Clinical Institute Withdrawal Assessment for Alcohol, Revised (CIWA-Ar). All clinical assessments were administered by trained psychiatrists according to standardized procedures.

### 2.4 Cognitive assessment

Cognitive function was evaluated by trained psychiatrists using a battery of standardized neuropsychological tests. Global cognitive function was assessed using the Mini-Mental State Examination (MMSE). Executive function was evaluated using the Modified Wisconsin Card Sorting Test (M-WCST), which provides measures including Response Correct (RC), Response Errors (RE), Perseverative Responses Errors(RPE), Non-Perseverative Errors (NRPE), and Categories Completed (CC). Verbal executive function was assessed using the Verbal Fluency Test (VFT).

All cognitive assessments were administered at baseline for both patients and healthy controls. For the longitudinal abstinence group, cognitive testing was repeated after four weeks of inpatient abstinence under the same testing conditions.

### 2.5 proBDNF measurement

Peripheral venous blood samples were collected in the morning after overnight fasting using EDTA anticoagulant tubes. Samples were centrifuged at 1,500 × g for 15 minutes at 4 °C, and plasma was aliquoted and stored at −80 °C until analysis. Plasma proBDNF concentrations were measured using a commercially available enzyme-linked immunosorbent assay (ELISA) kit (DuoSet Human proBDNF ELISA Development System, R&D Systems, USA) according to the manufacturer’s instructions. All samples were measured in duplicate, and the mean value was used for analysis. The standard curve was generated using a four-parameter logistic regression model, and concentrations were calculated based on optical density readings.

### 2.6 Statistical analysis

SPSS 25.0 software was used for statistical analysis, and GraphPad Prism 8.0 was used for generating figures. Continuous variables were tested for normality prior to analysis. Data that conformed to a normal distribution were represented by mean ± standard deviation(SD), and data that did not conform to a normal distribution were represented by median (interquartile range, P25–P75). Chi-square test was used for categorical variables. Independent samples t-test or nonparametric test was used for comparison between two groups. The paired samples Wilcoxon signed-rank test was used for comparison of continuous variables before and after treatment. Spearman correlation analysis was used for correlation of clinical variables. All statistical tests were two-tailed, and statistical significance was set at *P* < 0.05. Multivariable linear regression analyses were performed to examine whether plasma proBDNF levels were independently associated with executive function after adjusting for age, education level, and duration of alcohol use. In addition, logistic regression analysis was conducted to evaluate the association between plasma proBDNF levels and the likelihood of exhibiting lower executive performance (defined as scoring below the intra-sample median on the M-WCST Categories Completed (CC) score).

## 3 Results

### 3.1 Demographic and clinical characteristics

A total of 80 patients with alcohol dependence and 42 healthy controls were included in the present study. All participants were male and of Han ethnicity. There was no significant difference in age between the alcohol dependence group and the healthy control group (*P* = 0.325). The distribution of educational levels differed between groups, characterized by a higher proportion of participants educated beyond high school level in the healthy control group. Clinical characteristics related to alcohol use in the patient group, including duration of alcohol consumption and average daily alcohol intake, are summarized in **Table 1**. Data are presented as medians unless otherwise indicated.

**Table 1:** Baseline demographic and clinical characteristics of study participants.

Variable	Alcohol dependence group (n = 80)	Healthy controls (n = 42)	Statistic	P value
Age, years	42.5 (36.0–48.8)	38.0 (29.8–50.0)	Z=-0.984	0.325
Education, n (%)	χ² = 1.369	0.171		
- High school or below	35 (43.8%)	13 (31.0%)	—	—
- Above high school	45 (56.2%)	29 (69.0%)	—	—
Duration of drinking, years	19.0 (10.3–22.8)	—	—	—
Average daily ethanol intake, g/day	208 (208–246)	—	—	—
AUDIT score	26.5 (22–32)	—	—	—
SADQ score	28.8 (22–34)	—	—	—
CIWA-Ar score	11.5 (6–18)	—	—	—

### 3.2 Plasma proBDNF levels in patients and controls

**Figure 1:**
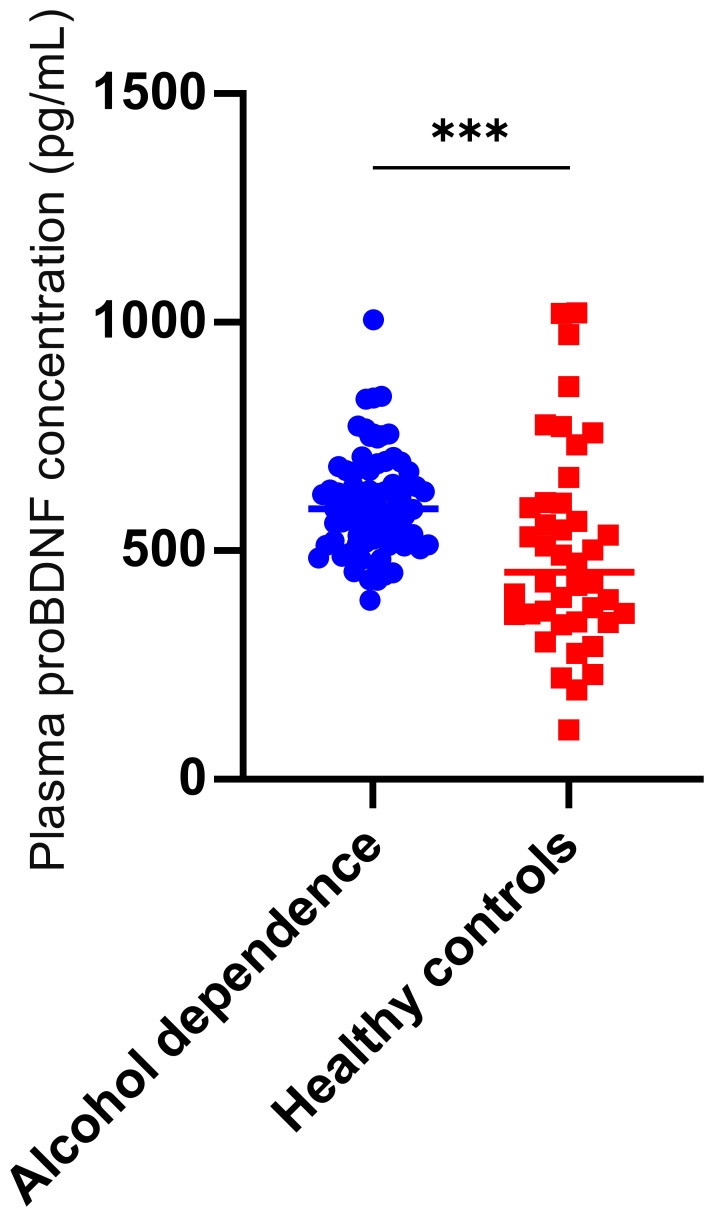
Plasma proBDNF levels in patients with alcohol dependence and healthy controls. Plasma proBDNF concentrations were significantly higher in patients with alcohol dependence compared with healthy controls. Data are presented as medians with interquartile ranges. Statistical analysis was performed using the Mann–Whitney U test. ***P < 0.001.

Plasma proBDNF concentrations were quantified in patients with alcohol dependence and healthy controls. Individual data points and group distributions are illustrated in **Figure 1**. A marked difference in plasma proBDNF levels was observed between the two groups. As shown in **Figure 1**, the median plasma proBDNF concentrations was 591.7 pg/mL (interquartile range [IQR], 522.7–672.2 pg/mL) in patients with alcohol dependence, which was significantly higher than those observed in healthy controls (452.7 pg/mL [IQR, 361.2–601.7 pg/mL]; Mann–Whitney U test, *P* < 0.001). This robust elevation of circulating proBDNF levels in the patient group indicates a marked disease-related alteration.

Inspection of the scatter plot demonstrates that plasma proBDNF concentrations in patients with alcohol dependence were broadly distributed, with the majority of values clustering at higher levels relative to healthy controls. In contrast, proBDNF concentrations in the healthy control group were generally lower and displayed a comparatively narrower distribution. Although some degree of inter-individual variability was present in both groups, the overall separation between patients and controls was evident, with minimal overlap between the interquartile ranges of the distributions. The group difference in plasma proBDNF concentrations was not attributable to demographic imbalance. As described in Section 3.1, patients and healthy controls were comparable in age, and all participants were male and of the same ethnic background. Therefore, the observed elevation of plasma proBDNF in patients with alcohol dependence likely reflects disease-related alterations rather than confounding demographic factors.

### 3.3 Cognitive performance differences between groups

Cognitive performance was compared between patients with alcohol dependence and healthy controls using a battery of standardized neuropsychological tests. The results demonstrated significant impairments across multiple cognitive domains in the alcohol dependence group, as summarized in **Table 2**. Patients with alcohol dependence showed significantly lower scores on the Mini-Mental State Examination (MMSE) compared with healthy controls, indicating reduced global cognitive function. In addition to overall cognitive impairment, deficits were particularly evident in executive function measures derived from the Modified Wisconsin Card Sorting Test (M-WCST).

**Table 2:** Comparison of cognitive performance between patients with alcohol dependence and healthy controls.

Variable	Alcohol dependence (n = 80)	Healthy controls (n = 42)	Statistic (Z)	P value
MMSE score	27.0 (25.3–28.0)	29.5 (29.0–30.0)	-7.333	<0.001
M-WCST Response Correct (RC)	30 (24–33)	32 (26–37)	-2.099	0.036
M-WCST Response Errors (RE)	19.0 (15.0–24.0)	14.0 (11.0–18.0)	-4.742	<0.001
M-WCST Non-Perseverative Errors (NRPE)	9.5 (6–12.8)	6 (3.8–8.0)	-4.161	<0.001
M-WCST Categories Completed (CC)	4 (2–5)	5 (3–6)	-2.228	0.026
Verbal Fluency Test (VFT) score	15 (13–20)	21.5 (16–26)	-4.307	<0.001

Specifically, patients with alcohol dependence exhibited a reduced number of correct responses and completed fewer categories on the M-WCST relative to healthy controls. Conversely, the numbers of Response Errors and Non-Perseverative Errors were significantly higher in the patient group, reflecting impaired cognitive flexibility and reduced efficiency in problem-solving strategies. These findings indicate marked executive impairment in individuals with alcohol dependence. Consistent with the observed executive deficits, performance on the Verbal Fluency Test (VFT) was also significantly poorer in patients with alcohol dependence than in healthy controls. Reduced verbal fluency further suggests impairments in executive control processes, including cognitive retrieval speed and strategic search abilities, which are commonly affected by chronic alcohol exposure.

Taken together, these results demonstrate that patients with alcohol dependence exhibit widespread cognitive impairments compared with healthy controls, with prominent deficits in global cognition and executive function. The pattern of impairments observed across MMSE, M-WCST, and VFT measures highlights executive impairment as a core cognitive feature of alcohol dependence. These group differences provide a functional context for subsequent analyses examining the relationships between cognitive performance and plasma proBDNF levels.

### 3.4 Correlations between proBDNF and alcohol-related variables

**Figure 2:**
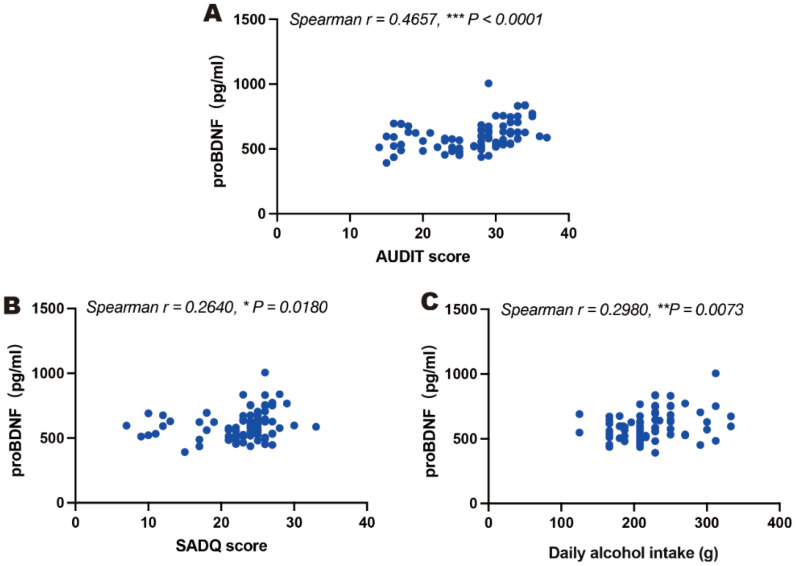
Correlations between plasma proBDNF levels and alcohol-related variables in patients with alcohol dependence. Scatter plots illustrate the relationships between plasma proBDNF concentrations and **(A)** Alcohol Use Disorders Identification Test (AUDIT) scores, **(B)** Severity of Alcohol Dependence Questionnaire (SADQ) scores, and **(C)** average daily alcohol intake. Correlation analyses were performed using Spearman’s rank correlation coefficient. Each data point represents an individual participant. *P < 0.05, **P < 0.01, ***P < 0.001.

To investigate the associations between plasma proBDNF levels and alcohol-related clinical characteristics, correlation analyses were performed in patients with alcohol dependence using Spearman’s rank correlation coefficient. The results are summarized in **Figure 2**. ProBDNF levels were positively correlated with alcohol use severity as measured by the Alcohol Use Disorders Identification Test (AUDIT), as shown in **Figure 2A**. Higher plasma proBDNF concentrations were associated with higher AUDIT scores, indicating a moderate positive correlation between circulating proBDNF levels and alcohol use behaviors (Spearman’s *r* = 0.466, *P* < 0.0001). In addition, plasma proBDNF levels showed a significant positive correlation with the Severity of Alcohol Dependence Questionnaire (SADQ) scores. Patients with higher proBDNF concentrations tended to exhibit greater dependence severity, although the strength of this association was weaker than that observed for AUDIT scores (Spearman’s *r* = 0.264, *P* = 0.018; **Figure 2B**). Plasma proBDNF levels were also positively correlated with average daily alcohol intake. Individuals reporting higher levels of daily alcohol consumption demonstrated increased circulating proBDNF concentrations, as shown in **Figure 2C** (Spearman’s r = 0.298, *P* = 0.007). This finding suggests a relationship between plasma proBDNF levels and cumulative alcohol exposure. Taken together, these results indicate that elevated plasma proBDNF levels are consistently associated with greater alcohol use severity, dependence severity, and daily alcohol consumption in patients with alcohol dependence.

### 3.5 Associations between proBDNF and cognitive function

**Figure 3:**
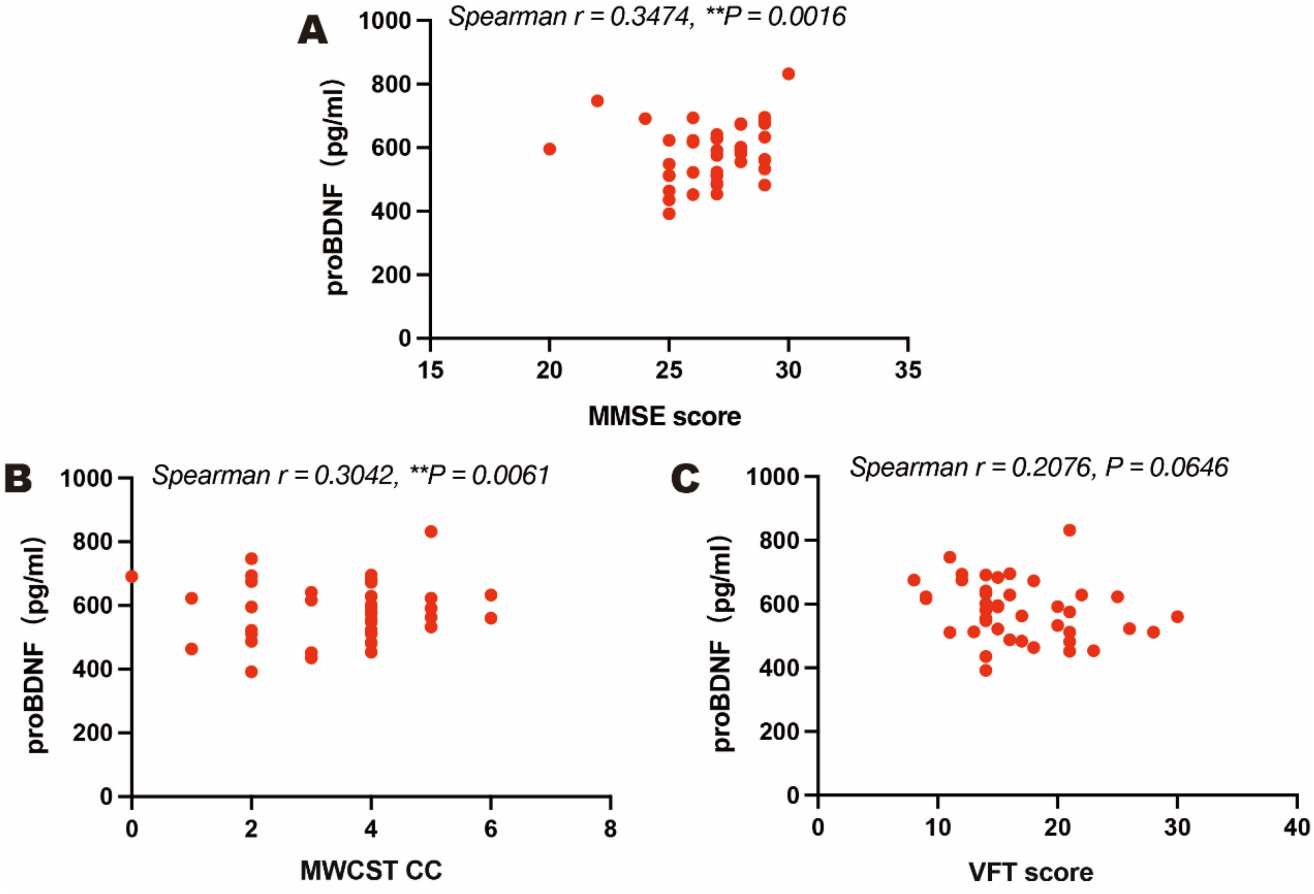
Correlations between plasma proBDNF levels and cognitive performance in patients with alcohol dependence. Scatter plots illustrate the relationships between plasma proBDNF concentrations and **(A)** MMSE scores, **(B)** Categories Completed (CC) score on the M-WCST, and **(C)** VFT scores. Correlation analyses were performed using Spearman’s rank correlation coefficient. Each data point represents an individual participant.** *P* < 0.01.

To further investigate the relationship between plasma proBDNF levels and cognitive function, correlation analyses were conducted between plasma proBDNF levels and neuropsychological test performance in patients with alcohol dependence. Spearman’s rank correlation coefficient was used for all analyses. The results are presented in **Figure 3**. Plasma proBDNF levels were negatively correlated with global cognitive performance as assessed by the MMSE. Higher plasma proBDNF concentrations were associated with lower MMSE scores, indicating poorer overall cognitive function in patients with elevated circulating proBDNF levels (Spearman’s *r* = 0.347, *P* = 0.002; **Figure 3A**). In analyses of executive function, plasma proBDNF levels showed a significant negative correlation with the Categories Completed (CC) score on the M-WCST. Patients with higher plasma proBDNF concentrations tended to complete fewer categories, reflecting impaired cognitive flexibility and reduced executive control, as shown in **Figure 3B** (Spearman’s *r* = 0.304, *P* = 0.006). Similarly, plasma proBDNF levels were negatively correlated with VFT scores. Increased circulating proBDNF concentrations were associated with poorer verbal fluency performance, further supporting a link between elevated proBDNF levels and executive impairment, as shown in **Figure 3C** (Spearman’s *r* = 0.208, *P* = 0.065). Taken together, these findings demonstrate that higher plasma proBDNF levels are associated with poorer cognitive performance across multiple domains in patients with alcohol dependence.

### 3.6 Multivariable regression analyses of lower executive performance

To further examine whether plasma proBDNF levels were independently associated with executive performance, multivariable regression analyses were performed in patients with alcohol dependence (**Tables 3**, **4**).

**Table 3:** Multivariable linear regression analysis for executive function (M-WCST categories completed).

Variable	β (SE)	Standardized β	P value
proBDNF (pg/mL)	0.003 (0.002)	0.21	0.052
Age (years)	−0.089 (0.028)	−0.41	0.002
Education (years)	0.067 (0.091)	0.09	0.461
Duration of alcohol use (years)	0.040 (0.031)	0.16	0.205

F = 4.28, adjusted R² = 0.15, P = 0.003.

**Table 4:** Logistic regression analysis for lower executive performance.

Variable	Odds ratio (95% CI)	P value
proBDNF (pg/mL)	1.004 (1.000–1.008)	0.030
Age (years)	1.12 (1.03–1.23)	0.012
Education (years)	0.94 (0.82–1.08)	0.381
Duration of alcohol use (years)	1.02 (0.97–1.08)	0.402

Likelihood ratio χ² = 18.6, P = 0.0015.

In multivariable linear regression analyses adjusting for age, education level, and duration of alcohol use, plasma proBDNF levels showed a borderline positive trend with executive function, as measured by the Categories Completed (CC) score on the Modified Wisconsin Card Sorting Test (M-WCST) (*β* = 0.003, *P* = 0.052). Age remained a significant independent predictor of executive function (*β* = -0.089, *P* = 0.002), whereas education level and duration of alcohol use were not independently associated with M-WCST performance. Intriguingly, when evaluating executive performance as a binary outcome based on the intra-sample median, the logistic regression analysis yielded a distinct localized pattern. After controlling for the same covariates, higher plasma proBDNF levels were independently associated with a marginal increase in the likelihood of scoring below the median on the M-WCST CC score (odds ratio = 1.004, *P* = 0.030).

### 3.7 Changes after four weeks of abstinence

**Figure 4:**
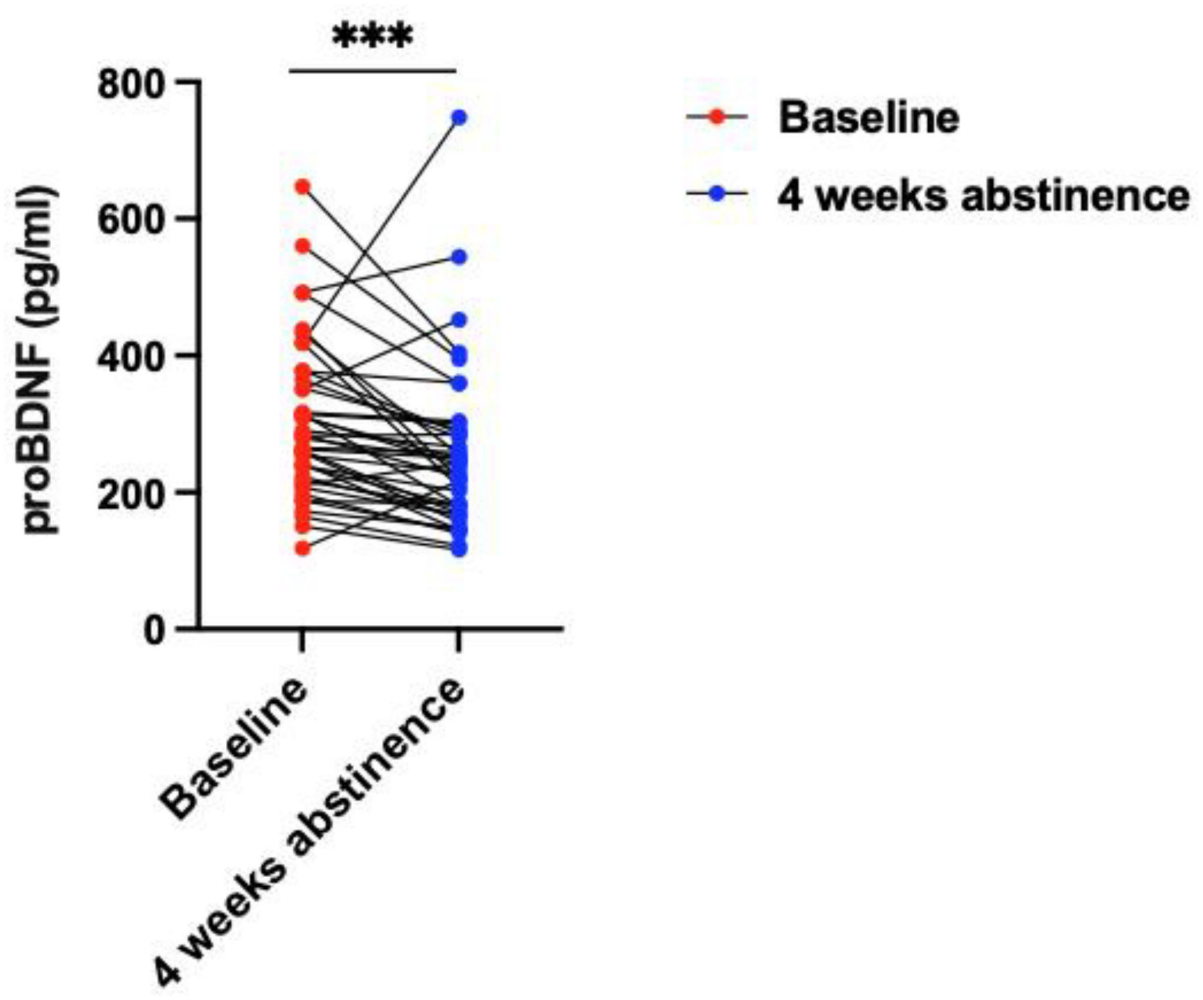
Changes in plasma proBDNF levels after four weeks of abstinence. Plasma proBDNF concentrations measured by ELISA at baseline and after four weeks of abstinence in patients with alcohol dependence. Lines connect individual data points across the two time points. Paired comparisons were performed using the Wilcoxon signed-rank test. ***P < 0.001.

To evaluate the effects of short-term abstinence on biological and cognitive outcomes, changes in plasma proBDNF levels and cognitive performance were examined after four weeks of abstinence in patients with alcohol dependence. Paired comparisons were performed using the Wilcoxon signed-rank test. The results are summarized in **Figure 4** and **Table 5**. Plasma proBDNF concentrations showed a significant reduction after four weeks of abstinence compared with baseline levels, as measured by ELISA (**Figure 4**). Most participants exhibited a decrease in circulating proBDNF levels following abstinence, indicating a consistent trend toward normalization during early abstinence. In parallel, cognitive performance improved significantly after four weeks of abstinence (**Table 5**). The Mini-Mental State Examination (MMSE) scores, reflecting global cognitive function, were significantly higher at follow-up than at baseline. Measures of executive function derived from the Modified Wisconsin Card Sorting Test also showed significant improvement, including increased numbers of Response Correct (RC) and Completed Categories (CC), along with reductions in Response Errors (RE) and Non-Perseverative Errors (NPE). Consistent with these findings, performance on the Verbal Fluency Test was significantly enhanced after four weeks of abstinence. In addition, qPCR analysis revealed significant changes in the expression of several genes related to BDNF signaling after four weeks of abstinence (**Supplementary Table 1**). Together, these results indicate that short-term abstinence is associated with both a reduction in plasma proBDNF levels and a concomitant improvement in cognitive function in patients with alcohol dependence.

**Table 5:** Changes in cognitive performance after four weeks of abstinence.

Variable	Baseline	4 weeks abstinence	Statistic	P value
MMSE score	26.0 (24.0–28.0)	27.0 (25.0–29.0)	−4.12	<0.001
M-WCST RC	24.0 (19.0–30.0)	30.0 (22.0–36.0)	−4.35	<0.001
M-WCST RE	22.0 (17.0–29.0)	18.0 (12.0–24.0)	−4.01	<0.001
M-WCST NRPE	12.0 (8.0–17.0)	9.0 (5.0–14.0)	−3.09	0.002
M-WCST CC	3.0 (2.0–4.0)	4.0 (3.0–5.0)	−4.27	<0.001
VFT score	15.0 (12.0–20.0)	18.0 (14.0–23.0)	−2.88	0.004

## 4 Discussion

In the present study, we systematically investigated alterations in plasma proBDNF levels in patients with alcohol dependence and examined their associations with alcohol-related severity, cognitive function, and short-term abstinence. Several important findings emerged. Plasma proBDNF levels were significantly elevated in patients with alcohol dependence compared with healthy controls, and higher proBDNF concentrations were associated with greater alcohol use severity and more pronounced cognitive impairment. Furthermore, plasma proBDNF levels decreased after four weeks of abstinence, accompanied by partial improvement in cognitive performance. Together, these findings suggest that dysregulation of proBDNF is closely linked to both the clinical severity and neurocognitive consequences of alcohol dependence and that such alterations may be, at least in part, reversible during early abstinence (22).

Accumulating evidence indicates that the biological functions of BDNF are highly dependent on its molecular form. BDNF is synthesized as a precursor protein, proBDNF, which is subsequently cleaved to produce mature BDNF (mBDNF). These two forms exert distinct and often opposing effects on neuronal structure and synaptic function. While mBDNF preferentially activates TrkB receptors to promote synaptic strengthening, neuronal survival, and long-term potentiation, proBDNF primarily signals through the p75 neurotrophin receptor (p75NTR), often in combination with sortilin, to facilitate synaptic weakening, long-term depression, and synaptic pruning (13, 18, 28). Experimental studies have demonstrated that activation of proBDNF–p75NTR signaling suppresses hippocampal neurogenesis, inhibits synaptic plasticity, and impairs learning and memory processes (20). Therefore, an imbalance favoring proBDNF over mBDNF is increasingly recognized as a critical mechanism underlying cognitive impairment in various neuropsychiatric and neurodegenerative conditions (13, 28).

Cognitive impairment is a well-established consequence of chronic alcohol use, with executive impairment representing one of the most consistently affected domains. Executive functions, including cognitive flexibility, inhibitory control, and verbal fluency, rely heavily on the integrity of prefrontal–striatal and prefrontal–hippocampal circuits, which are particularly vulnerable to alcohol-related neurotoxicity (8). The M-WCST was selected for this study as it eliminates ambiguous sorting cards and reduces testing fatigue while maintaining high sensitivity to frontal lobe deficits (29).In the present analysis, our multivariable models revealed a nuanced, divergent pattern regarding peripheral proBDNF: while the linear model indicated a borderline positive trend with continuous M-WCST CC scores (*β* = 0.003), the median-split logistic model suggested a marginal risk correlation (OR = 1.004). This subtle divergence underscores the complexity of evaluating circulating neurotrophic precursors and likely reflects the distinct statistical sensitivity of different modeling approaches. Physiologically, peripheral plasma proBDNF levels are subject to systemic clearance and metabolic variances during early abstinence, which may not linearly mirror central prefrontal dynamics. Furthermore, because an intra-sample median split inherently reduces statistical power and introduces artificial thresholds, these borderline, exploratory findings must be interpreted with strict caution. Rather than demonstrating a definitive pathological trajectory, our results highlight a complex, non-linear equilibrium within the peripheral neurotrophin system in addiction biology. This variance underscores the critical need for future large-scale, prospective studies with concurrent mBDNF assays to clarify whether these observations reflect a transient adaptive stage or statistical artifacts within a limited sample. Our results also extend prior clinical observations by suggesting that proBDNF, rather than total BDNF alone, may be more closely linked to cognitive impairment in alcohol dependence. This distinction may help reconcile discrepancies in the literature and underscores the relevance of examining specific components of the BDNF system (4, 13).

An additional strength of this study is the longitudinal assessment of changes following short-term abstinence. After four weeks of abstinence, plasma proBDNF levels were significantly reduced, and cognitive performance showed measurable improvement across multiple domains. These findings suggest that proBDNF dysregulation in alcohol dependence is dynamic and partially reversible during early abstinence. However, the absence of a continued-drinking control group—precluded by ethical imperatives in a clinical setting—limits our ability to isolate proBDNF reductions from potential natural biological fluctuations. Thus, these longitudinal findings should be interpreted with caution. Nevertheless, cognitive recovery was incomplete, indicating that normalization of circulating proBDNF levels does not directly translate into full restoration of cognitive function. Persistent synaptic alterations, structural brain changes, and long-lasting disruptions in neurotrophic signaling may contribute to delayed or incomplete recovery. These observations are consistent with previous reports showing that cognitive deficits may persist for months or even years after cessation of alcohol use (27).

From a clinical perspective, the observed associations between plasma proBDNF levels, alcohol use severity, cognitive impairment, and abstinence-related changes suggest that proBDNF may serve as a potential biomarker reflecting disease burden and early biological response to abstinence. However, several caveats should be considered. Circulating proBDNF levels may be influenced by peripheral sources and systemic factors, and their relationship to central nervous system processes remains indirect. Moreover, the lack of concurrent measurement of mBDNF in the present study precludes direct evaluation of the balance between precursor and mature forms, which may be critical for interpreting the functional significance of altered proBDNF levels. Additionally, the screening of healthy controls relied on clinical interviews and routine markers (GGT/MCV). Despite normal ranges, the potential for recall bias remains, and the lack of highly specific biomarkers like Carbohydrate-Deficient Transferrin (CDT) means we cannot entirely exclude occult alcohol use, which might account for the variance observed in a subset of the control group. The confounding influence of standard interventions—including thiamine, benzodiazepine tapering (7–10 days), and group psychotherapy—must also be acknowledged, as these may have modulated the recovery trajectories of both proBDNF and cognition. Finally, our relatively homogeneous sample of Han Chinese men aged 18–55 years reduced heterogeneity related to sex, age range, ethnicity, and treatment setting; however, this also limits the generalizability of the findings to women, older adults, other ethnic groups, and patients treated in different clinical settings. Future multi-center studies with more diverse cohorts are necessary to confirm the universality of these findings. Future studies integrating longitudinal measurements of both proBDNF and mBDNF, together with neuroimaging and detailed cognitive assessments, are warranted to further elucidate the clinical and biological relevance of BDNF system dysregulation in alcohol dependence.

## Data Availability

The original contributions presented in the study are included in the article/**Supplementary Material**. Further inquiries can be directed to the corresponding author/s.
