# Supplementary material for: Association between plasma proBDNF levels and cognitive impairment in patients with alcohol dependence: a case–control and longitudinal study
Source: Front Psychiatry. 2026 Jun 10;17:1835592. doi: 10.3389/fpsyt.2026.1835592 (PMC13290814; doi:10.3389/fpsyt.2026.1835592)
Supplement: Supplementary file 1 [file Table1.docx]

**Supplementary Table S1. Changes in gene expression related to BDNF signaling after four weeks of abstinence**

| **Gene** | **Baseline** | **4weeks abstinence** | **Statistic (Wilcoxon Z)** | **P value** |
| --- | --- | --- | --- | --- |
| BDNF | 0.00436 (0.00243–0.01069) | 0.01048 (0.00274–0.02632) | −3.92 | <0.001 |
| p75NTR (NGFR) | 0.0027 (0.0008–0.0041) | 0.0013 (0.0003–0.0019) | −3.85 | <0.001 |
| Sortilin (SORT1) | 0.0464 (0.0321–0.0706) | 0.0385 (0.0257–0.0643) | −2.74 | 0.006 |
| PAI-1 (SERPINE1) | 0.0181 (0.0078–0.0332) | 0.0123 (0.0039–0.0243) | −3.21 | 0.001 |
| tPA (PLAT) | 0.0036 (0.0012–0.0071) | 0.0021 (0.0005–0.0041) | −2.68 | 0.007 |
| TrkB (NTRK2) | 0.00061 (0.00014–0.00142) | 0.00026 (0.00007–0.00037) | −3.47 | <0.001 |

Data are presented as median (interquartile range). Gene expression levels were calculated using the 2^−ΔCt method and compared between baseline and four weeks of abstinence using the Wilcoxon signed-rank test.

Gene expression levels were measured by quantitative real-time PCR at baseline and after four weeks of abstinence in patients with alcohol dependence. Data are presented as median (interquartile range). Paired comparisons were performed using the Wilcoxon signed-rank test.
